# Reporting quality of abstracts of systematic reviews/meta-analyses: An appraisal of Arab Journal of Urology across 12 years: the PRISMA-Abstracts checklist

**DOI:** 10.1080/2090598X.2022.2113127

**Published:** 2022-08-22

**Authors:** Walid El Ansari, Khalid AlRumaihi, Kareem El-Ansari, Mohamed Arafa, Haitham Elbardisi, Ahmad Majzoub, Ahmad Shamsodini, Abdulla Al Ansari

**Affiliations:** a Department of Surgery, Hamad Medical Corporation, Doha, Qatar; b College of Medicine, Qatar University, Doha, Qatar; c Weill Cornell Medicine – Qatar, Doha, Qatar; d Urology Department, Hamad Medical Corporation, Doha, Qatar; e Faculty of Medicine, Ain Shams University, Cairo, Egypt; f Andrology Department, Cairo University, Cairo, Egypt

**Keywords:** Reporting quality, systematic review, Meta-analysis, urology, abstract, PRISMA-abstract

## Abstract

**Objective:**

We appraised the reporting quality of abstracts of systematic reviews/meta-analyses (SR/MAs) published in one urology journal and explored associations between abstract characteristics and completeness of reporting.

**Methods:**

The Arab Journal of Urology (AJU) was searched for SR/MAs published between January 2011 and 31 May 2022. SR/MAs with structured abstract and quantitative synthesis were eligible. Two reviewers simultaneously together selected the SR/MAs by title, screened the abstracts, and included those based on inclusion/exclusion criteria. Data of a range of characteristics were extracted from each SR/MAs into a spreadsheet. To gauge completeness of reporting, the PRISMA-Abstract checklist (12 items) was used to appraise the extent to which abstracts adhered to the checklist. For each abstract, we computed item, section, and overall adherence. Chi-square and t-tests compared the adherence scores. Univariate and multivariate analyses identified the abstract characteristics associated with overall adherence.

**Results:**

In total, 66 SR/MAs published during the examined period; 62 were included. Partial reporting was not uncommon. In terms of adherence to the 12 PRISMA-A items were: two items exhibited 100% adherence (title, objectives); five items had 80% to <100% adherence (interpretation, included studies, synthesis of results, eligibility criteria, and information sources); two items displayed 40% to <80% adherence (description of the effect, strengths/limitations of evidence); and three items had adherence that fell between 0% and 1.6% (risk of bias, funding/conflict of interest, registration). Multivariable regression revealed two independent predictors of overall adherence: single-country authorship (i.e. no collaboration) was associated with higher overall adherence (*P* = 0.046); and abstracts from South America were associated with lower overall adherence (*P* = 0.04).

**Conclusion:**

This study is the first to appraise abstracts of SR/MAs in urology. For high-quality abstracts, improvements are needed in the quality of reporting. Adoption/better adherence to PRISMA-A checklist by editors/authors could improve the reporting quality and completeness of SR/MAs abstracts.

## Introduction

Urology is a rapidly expanding field, with many new management approaches and innovative technologies. To appraise such advances, compare the effectiveness of procedures and their side effects, or alternatively, to evaluate etiology, estimate prevalence, assess diagnosis or prognosis, systematic reviews/meta-analyses (SRs/MAs) are considered as a reliable source of information and evidence [1]. SRs/MAs assume the top position pertaining to the quality and dependability of the evidence generated, and are a reliable information source [2]. Combining the results of trials into a single SR integrates high-quality evidence [3]. Likewise, MAs of randomized-controlled trials with low risk of bias comprise high-level evidence of outcomes/consequences of interventions [4].

Time-pressed clinicians scan journal abstracts to judge the relevance of the research to their clinical practice, prior to retrieving the full-text, as abstracts do not replace the necessity to read the full articles [5]. Researchers, clinicians, policymakers, and research consumers depend on the knowledge presented in abstracts of SRs/MAs [6] as an initial step. For busy readers or those with no/limited access to full-texts, the stand-alone abstract needs to portray unambiguous summaries of the methods, results, and conclusions that correctly reveal the elements of the full-blown research [7].

The SR/MA’s abstract needs to spell out its systematic methodology [7]. Although structured abstracts are recognized and implemented by many journals; however, the quality of abstracts needs improvements [8]. Hence, reporting benchmarks can guarantee well-grounded judgements of the methodological quality of SRs [3] or MAs.

In urology, the Preferred Reporting Items for Systematic Reviews and Meta-Analyses (PRISMA) checklist [9] has been employed in many SRs/MAs, e.g. testicular tumours and azoospermia [10], artificial intelligence in endourology [11], minimally invasive treatment of urethral stricture [12], or coffee consumption and prostate cancer [13]. Fortunately, the reporting and methodological qualities of recently published MAs in paper-based urology journals have been generally good [14]. Notwithstanding, analysis of 227 published pediatric urology SRs/MAs upon which guidelines are premised found that many had poor methodology and, to a lesser extent, poor reporting quality [15]. In agreement, an appraisal of the SRs/MAs in pediatric urology journals reported that almost half lacked good scientific quality, raising concerns about their role in clinical practice [16]. However, the authors appraised the full text of the SRs/MAs rather than the abstracts [16].

To our knowledge, the reporting quality of abstracts of urological SRs/MAs has not been assessed to date. This is despite that: 1) abstracts are the most read portion of a medical article, and hence, need to provide clear-cut summaries of the methods, results, and conclusions that precisely display the ingredients and qualities of the full-blown research [7]; 2) research findings not published in English may have only the abstract in English, rendering it the sole information accessible to readers [7]; and, 3) a checklist is available as a reporting guideline for SRs/MAs abstracts [7]. Notably, assessments of the completeness of reporting of SRs/MAs abstracts have been undertaken in other fields e.g. dental specialties [3,5], anesthesiology [17], or general medical literature [18,19].

Therefore, the current study assessed the reporting quality of abstracts of SRs/MAs published in one prestigious peer-reviewed Pubmed-cited urology journal. We employed the PRISMA for Abstracts (The Preferred Reporting Items for Systematic Reviews and Meta-Analyses, PRISMA-A) Checklist [7] to appraise SRs/MAs published across 11 years. PRISMA primarily focuses on the reporting of reviews that evaluate the effects of interventions, but can also be used for reporting SRs that evaluate etiology, prevalence, diagnosis, or prognosis [20]. The current study is the first to undertake such in-depth appraisal of the quality of abstracts of SR/MAs in urology. The findings will contribute to the very thin evidence base to inform journals and editorial teams, health-care providers, authors, and funding agencies.

## Methods

### Setting: The Arab Journal of Urology (AJU)

The *AJU* publishes peer-reviewed open-access research and clinical material on all aspects of urology to the widest possible urological community worldwide. It is a viable international forum for the practical, timely, and state-of-the-art clinical urology and basic urological research [21]. The journal was selected as it is only one in the Middle East region that specializes in the field of urology. Over the last decade, the *AJU* witnessed a series of developments and advancements that resulted in its successful inclusion in Medline. It is now a Q2 journal (Q2, 2020, CiteScore Best Quartile), with impact 2.64 and the cite score of 5.1 (2021, CiteScore, Scopus) and ranks 21 out of 99 urology journals (79th percentile) [22]. The journal publishes manuscripts from all over the world, has endorsed the PRISMA guidelines, and specifies a structured abstract of a maximum of 300 words [21].

### Definitions

In line with assessments of the quality of SR/MA abstracts [23], SR was defined as a study that summarizes evidence from multiple studies with explicit reporting of methods; it aims to identify, critically appraise, and summarize evidence relating to a particular problem in an unbiased and systematic manner [24]. Meta-analysis is the statistical analysis of a large collection of results from individual studies for the purpose of integrating the findings [25]; a quantitative, formal, and epidemiological study design used to systematically assess the results of previous research to derive conclusions about that body of research [26].

### Research questions

The research questions were: ‘How complete is the reporting of abstracts of SRs/ MAs published in the *AJU*?’; ‘What are the common reasons behind partial reporting?’; ‘What are the levels of item, section and overall adherence?’ and, ‘Are there any associations between abstract characteristics and the completeness of reporting?’

### Data source and search strategy

We searched the *AJU* website for SR/MAs published from 1 January 2011 to 31 May 2022 and then updated the searching to 19 May 2022. We limited the time range because the first articles available online at the *AJU* website were in 2011 (2011, vol 9, issue 1). Articles published during this period with the term ‘systematic review’ or ‘systematic’ or ‘review’ or ‘meta-analysis’ or ‘metanalysis’ or ‘metaanalysis’ in their title, abstract, or full text, were eligible. A systematic search of tables of contents (TOC) of the *AJU* was conducted in duplicate by two authors (last inspection as of 19 May 2022). In order not to potentially miss any eligible article, after the electronic search of the TOC, all online volumes and issues were hand-searched to discover any article that fulfilled the inclusion criteria but did not include the search terms in the title.

### Eligibility criteria and study selection

Articles with the search terms in their title, abstract, or full text, were included. Inclusion criteria were that articles are in English, and those in which the authors explicitly declared an intention to conduct an SR (±MA). Only structured abstracts were included, and there was no limitation on the population, exposure/intervention, health issues, as well as study design of articles included in each SR. Exclusion criteria were all other study types other than SR/MAs, such as narrative reviews, descriptive studies (e.g. case reports, case series, and prevalence studies), analytic studies (e.g. cases and controls, cross-sectional and cohort), experimental studies (e.g. controlled and uncontrolled clinical trials), conference abstracts, or SR/MAs with qualitative synthesis or with unstructured abstracts. All eligible articles were selected by two reviewers working simultaneously together. The reviewers evaluated the titles for eligibility, and in cases of disagreement, consensus was reached through discussion.

### Training and data extraction

The following information was retrieved from the included abstracts: year/volume/issue of publication; first author’s country of affiliation; number of authors; whether the author team was from the same country or if there were collaborations with other countries; in the case of international collaborations, the country/ies was recorded; whether an MA was performed or not; research topic; number of words of the abstract; and number of studies/patients included. Several training sessions were provided on abstracts of SR/MAs from another journal that involved reading each PRISMA-A checklist item and discussing its meaning/purpose. Practical training involved application of the checklist to 10 abstracts that were not part of the studied sample. Although reviewers worked simultaneously together on each abstract, disagreements were resolved by discussion. An Excel file of the initial 66 titles was produced, all abstracts were coded for the presence/absence of a structured format, and unstructured abstracts were not subjected to further appraisal and were excluded.

### Assessment of abstract reporting: The PRISMA-A checklist

We appraised abstracts of SRs/ MAs published in the *AJU*, aiming to: 1) assess the completeness of reporting; 2) investigate the most common reasons behind partial reporting; and, 3) compute item, section and overall adherence. In addition, we explored the univariate/multivariate associations between overall adherence to PRISMA-A and seven abstract characteristics (number of authors, country, publication period, topic of study, collaboration, whether abstract was for SR or for SR+MA, and abstract word count). PRISMA-A was developed as an extension to the PRISMA statement, to provide focused guidance on writing abstracts for SR [7].

Table 1 shows the six PRISMA-A sections (title, objective, methods, results, discussion and other information), and the 12 items pertaining to different sections. In line with others who used a three-point scale to evaluate each item of the PRISMA-A [3], we employed the PRISMA-A checklist to appraise the SR/MAs abstracts, scoring the adherence of each SR/MAs abstract under examination to each PRISMA-A item on a 3-point scale (1 = item not reported at all, 2 = item partially reported, 3 = item fully reported). Hence, abstracts with higher scores were regarded as having better reporting, and the highest possible score was 36 points.

**Table 1.** PRISMA-A checklist: sections and items.

Section/ Item	Explanation/ Guidance
Title	
1. Title	Identify the report as a systematic review, meta-analysis, or both
Background	
2. Objectives	The research question including components (participants, interventions, comparators, outcomes)
Methods	
3. Eligibility criteria	Study and report characteristics used as criteria for inclusion
4. Information sources	Key databases searched and search dates
5. Risk of bias	Methods of assessing risk of bias
Results	
6. Included studies	Number and type of included studies and participants and relevant characteristics of studies
7. Synthesis of results	Results for main outcomes (benefits and harms), preferably indicating number of studies and participants for each. If MA was done, include summary measures and confidence intervals
8. Description of effect	Direction of effect (i.e. which group is favoured) and size of effect in terms meaningful to clinicians and patients
Discussion	
9. Strengths/ limitations of evidence	Brief summary of strengths and limitations of evidence (e.g. inconsistency, imprecision, indirectness, or risk of bias, other supporting or conflicting evidence)
10. Interpretation	General interpretation of the results and important implications
Other	
11. Funding	Primary source of funding for the review
12. Registration	Registration number and registry name

Beller et al 2013 [7]

### Statistical analysis

Data were presented as mean± standard deviation (SD) or frequency and percentage as appropriate. For comparisons of adherence to PRISMA-A, chi-square or Fisher’s exact test and t test as appropriate compared the categorical and continuous variables, respectively. Univariate regression and multivariate linear regression analyses identified the characteristics (independent variables) associated with the % of overall adherence (continuous dependent variable). Seven characteristics were included in the model: country of authorship (geographic zone), number of authors, research topic, meta-analysis or not, collaboration or not, publication period, and % of abstract word count used. ß coefficients denoting strength of the effect (change in the dependent variable i.e. % of overall adherence with each unit change in the independent variable/predictor) and their 95% confidence intervals (CI) were computed. Likewise, and R^2^ estimates (proportion of variance in the dependent variable that can be explained by the independent variables/predictors included in the linear regression models) were generated for each characteristic. Data analysis used the Statistical Package for Social Sciences v.21 (SPSS Inc., Chicago, IL), with significance (2-tailed) set at *P* < 0.05.

## Results

### Systematic reviews and meta-analyses included

**Figure 1.**
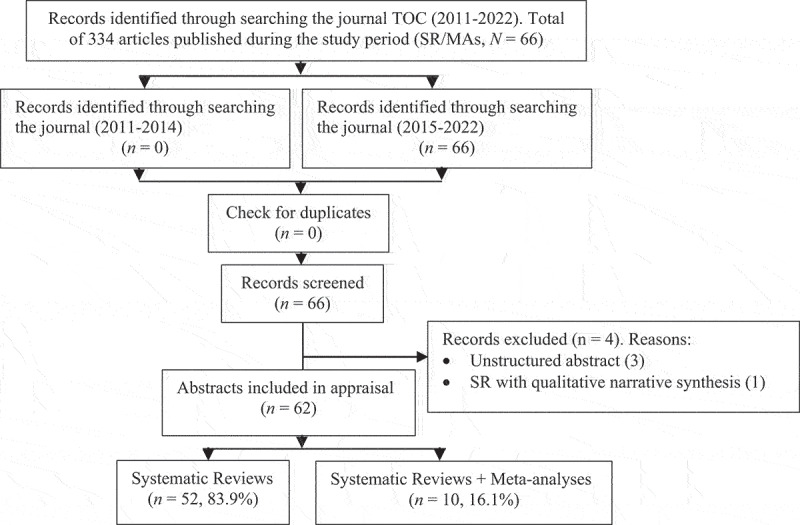
Study flowchart.

We assessed 66 abstract titles published during the period under examination. After reading the abstracts’ full text, four were excluded (Figure 1, study flowchart). The remaining 62 included in the analysis represented SRs (*n* = 52, 83.9%) or SR + MA (*n* = 10, 16.2%). Appendix 1 details the 66 abstracts published during the period examined.

### Characteristics: Frequencies, authorship, research topics, abstract word count, numbers of included studies and participants

*AJU* witnessed a boost in the numbers of published SR/MAs during the last two years (Table 2). There was a near-equal distribution of abstracts with 1–4 authors or ≥ 5 authors. More than half of the SR/MAs were related to treatment effects, while the remaining covered wider issues. Based on the first author’s affiliation, the UK, USA, and Egypt had the most SR/MAs; and Europe generally contributed about half of the SR/MAs. Slightly less than half of the SR/MAs were by authors from one country, while the remaining were collaborations between different nations. Collaborating authors were from one or more countries in Europe, Asia, Africa, and North America. Table 2 also shows the number of words as well as the percentage of the permitted abstract word count that were actually used by the authors. Fewer abstracts (14.5%) did not report the number of primary studies included in the SR/MAs; but more abstracts (72.6%) did not report the number of patients included.

**Table 2.** Selected characteristics of included abstracts.

Characteristic	Category	Value
Publication period	≥ 2020; ≤ 2019	28 (45.2%); 34 (54.8%)
Type	SR; SR+MA	52 (83.9%); 10 (16.1%)
Number of authors	Median; range	2; 1–16
	1–4 authors; ≥ 5 authors	32 (51.6%); 30 (48.4%)
Research topic	Treatment; Other *^a^*	32 (51.6%); 30 (48.4%)
First author affiliation	Country	UK (24.2%), USA (21%), Egypt (9.7%), Germany (6.5%); Malaysia, Qatar (4.8% each); Austria, Lebanon, Greece, Switzerland (3.2% each); France, Ireland, Hong Kong, India, Saudi Arabia, Bahrain, Colombia, Brazil, South Africa (1.6% each)
	Continent	Europe (n = 28, 45.2%); Africa (7, 11.3%); N America (13, 21%); S America (2, 3.2%); Asia (12,19.4%)
Collaboration *^b^*	No; Yes	27 (43.5%); 35 (56.5%)
	With 1 country; > 1 country	16 (25.8%); 19 (30.6%)
Collaborating	Europe	Netherlands, France, Italy, Poland, Czech Republic
continent/ countries	Asia	Jordan, UAE, Iran, India, Pakistan, Japan, Russia, China
	Africa	Algeria, Nigeria
	North America	Canada
Abstract word count	Mean ± SD; range	237.3 ± 46.1 words; 131–380 words
	≤ 250; > 250 words	40 (64.5%); 22 (35.5%). Exceeding word count *^c^* (n = 5, 8.1%)
	% of abstract word count used *^d^*	Range: 43.67–126.67%; ≤ 80% used (n = 32, 51.6%); > 80% used (30, 48.4%)
Primary studies	Number of studies	Not reported (n = 9, 14.5%) *^e^*; median 25; range: 1–339
	≤ 30; > 30 studies	34 (64.2%); 19 (35.8%)
	Number of patients	Not reported (n = 45, 72.6%) *^f^*; median 1630; range 194–450,367

SD standard deviation *^a^* Includes SR/MAs of e.g. current state, future prospects, updates, trends, clinical characteristics, roles, types and doses, indications, techniques and devices, incidence, diagnosis, evaluation and management, long-term consequences, complications, associations, etc.; *^b^* Whether all authors were from single country (No) or not (Yes); *^c^* Journal specifies 300-word word count for abstracts; *^d^* denotes % of the allowed abstract word count that was used by authors; *^e^* abstract did not report number of studies included; *^f^* abstract did not report number of patients included

### Completeness of reporting by PRISMA-A items

Nearly all abstracts fully reported the title and interpretation items (100% and 96.8%, respectively), while 54.8% fully reported the description of effect, and 38.7% fully reported the information sources (Table 3). Some items exhibited high partial reporting e.g. objectives (88.7% partially reported), eligibility criteria (75.8%), included studies (77.4%), and synthesis of results (79%). Risk of bias, funding, registration, and strengths/limitations of evidence were poorly reported.

**Table 3.** Completeness of reporting of PRISMA-A checklist items (N = 62).

	Category		
Item	Not reported	Partially reported	Fully reported
Title			
1. Title	0 (0)	0 (0)	62 (100)
Background			
2. Objectives	0 (0)	55 (88.7)	7 (11.3)
Methods			
3. Eligibility criteria	8 (12.9)	47 (75.8)	7 (11.3)
4. Information sources	9 (14.5)	29 (46.8)	4 (38.7)
5. Risk of bias	61 (98.4)	0 (0)	1 (1.6)
Results			
6. Included studies	7 (11.3)	48 (77.4)	7 (11.3)
7. Synthesis of results	7 (11.3)	49 (79)	6 (9.7)
8. Description of the effect	24 (38.7)	4 (6.5)	34 (54.8)
Discussion			
9. Strengths/ limitations of evidence	35 (56.5)	27 (43.5)	0 (0)
10. Interpretation	2 (3.2)	0 (0)	60 (96.8)
Other			
11. Funding and conflict of interest	62 (100)	0 (0)	0 (0)
12. Registration	62 (100)	0 (0)	0 (0)

All cell values represent n (%)

### Reasons for partial reporting by PRISMA-A items

Six PRISMA-A items had partial reporting ranging from 43.5% to 88.7% (Table 4). Partial reporting of objectives was mainly due to omissions in comparator, outcome/s, population, or a combination of population/comparator; omission of intervention was much less common. For the eligibility criteria, partial reporting was invariable because of omission of type of studies. In terms of included studies, partial reporting was most common due to omissions of type of studies and/or participants. As for information sources, omission of search dates was the common reason for its partial reporting. Partial reporting of synthesis of results was usually due to omission of the number of participants for each outcome alone or in combination with omission of the results for outcome (benefits and harms). In connection with strengths/limitations of evidence, partial reporting was because of omissions of strengths rather than limitations.

**Table 4.** Selected PRISMA-A items partially reported: frequency and reasons.

Item partially reported	N	%
Objectives	55	88.7
Missing sub-items		
Comparator (C)	13	**21**
Outcome/s (O)	9	**14.5**
Population (P)	8	**12.9**
P + C	8	**12.9**
P + O	5	8.1
O + C	5	8.1
C + Intervention (I)	2	3.2
O + P + C	2	3.2
P + C + I	2	3.2
P + I	1	1.6
Eligibility criteria	47	75.8
Missing sub-items		
Type of studies	47	**75.8**
Included studies	48	77.4
Missing sub-items		
Type of studies + participants	26	**41.9**
Type of studies	10	16.1
Participants	9	14.5
Number + type of studies	2	3.2
Number of studies + participants	1	1.6
Information sources	29	46.8
Missing sub-items		
Search dates	25	**40.3**
Name of database/s	4	6.5
Synthesis of results	49	79
Missing sub-items		
Number of participants for each outcome	24	**38.7**
Number of participants for each outcome + results for outcome (benefits & harms)	17	**27.4**
Number of studies	3	4.8
Results for outcome (benefits & harms)	1	1.6
Summary measures	1	1.6
CI	1	1.6
Number of studies + CI	1	1.6
Number of participants for each outcome + CI	1	1.6
Strengths/ limitations of evidence	27	43.5
Missing sub-items		
Strengths	22	**35.5**
Limitations	5	8.1

Bolded cells indicate the most common reasons for incomplete reporting for the given item; CI confidence interval

### Adherence to PRISMA-A

The mean overall adherence of the abstracts was 62.77 ± 9.5% (range = 33.3–75%). About 16% of abstracts had overall adherence of 30% to < 50%; 66% of abstracts exhibited overall adherence of >50% to < 75%; and 18% had 75% overall adherence. By section, Table 5 shows that mean adherence of the title and objectives sections was 100%; methods section was 58.06 ± 19.9; results and discussion sections were 79.57 ± 29.8 and 70.16 ± 24.7; while the ‘others’ section was 0%.

**Table 5.** Section and overall adherence* to PRISMA-A checklist by selected characteristics (N = 62).

Characteristic/ Section	Title	Background	Methods	Results	Discussion	Other	% Overall adherence
Whole Sample (n = 62)	100 ± 0	100 ± 0	58.1 ± 19.9	79.6 ± 29.8	70.2 ± 24.7	0 ± 0	62.8 ± 9.5
Number of authors							
1–4 (n = 32)	100 ± 0	100 ± 0	54.2 ± 23.6	72.9 ± 34.3	62.5 ± 22.0	0 ± 0	58.9 ± 10.4
≥ 5 (n = 30)	100 ± 0	100 ± 0	62.2 ± 14.5	86.7 ± 22.5	78.3 ± 25.2	0 ± 0	66.9 ± 6.4
*P*	**–**	**–**	NS	***0.034***	***0.005***	**–**	***0.001***
Publication period							
≤ 2019 (n = 34)	100 ± 0	100 ± 0	55.9 ± 22.8	71.6 ± 33.0	72.1 ± 25.1	0 ± 0	60.5 ± 10.5
≥ 2020 (n = 28)	100 ± 0	100 ± 0	60.7 ± 15.9	89.3 ± 22.3	67.9 ± 24.4	0 ± 0	65.5 ± 7.4
*P*	**–**	**–**	NS	***0.02***	NS	**–**	***0.04***
Collaboration							
Yes (n = 35)	100 ± 0	100 ± 0	56.2 ± 22.5	83.8 ± 20.4	67.1 ± 24.1	0 ± 0	62.9 ± 8.2
No (n = 27)	100 ± 0	100 ± 0	60.5 ± 16.1	74.1 ± 38.5	74.1 ± 25.5	0 ± 0	62.7 ± 11.2
*P*	**–**	**–**	NS	NS	NS	**–**	NS
Topic							
Treatment (n = 32)	100 ± 0	100 ± 0	54.2 ± 23.6	88.5 ± 21.8	68.8 ± 24.6	0 ± 0	63.8 ± 9.1
Other (n = 30)	100 ± 0	100 ± 0	62.2 ± 14.5	70 ± 34.3	71.7 ± 25.2	0 ± 0	61.7 ± 9.9
*P*	**–**	**–**	NS	***0.01***	NS	**–**	NS
Type							
SR (n = 52)	100 ± 0	100 ± 0	57.1 ± 21.2	75.6 ± 31.1	69.2 ± 24.6	0 ± 0	61.4 ± 9.6
SR + MA (n = 10)	100 ± 0	100 ± 0	63.3 ± 10.5	63.3 ± 10.5	75 ± 26.4	0 ± 0	70 ± 4.3
*P*	**–**	**–**	NS	NS	NS	**–**	***0.008***
Affiliation of first author: Geographical Zone							
Europe (n = 28)	100 ± 0	100 ± 0	59.5 ± 16.6	89.3 ± 22.3	76.8 ± 25.4	0 ± 0	66.7 ± 6.8
Africa (n = 7)	100 ± 0	100 ± 0	42.9 ± 31.7	80.9 ± 37.8	50 ± 0	0 ± 0	55.9 ± 9.3
N America (n = 13)	100 ± 0	100 ± 0	61.5 ± 18.5	66.7 ± 40.8	65.4 ± 24.0	0 ± 0	59.6 ± 11.7
S America (n = 2)	100 ± 0	100 ± 0	33.3 ± 47.1	66.7 ± 0	50 ± 0	0 ± 0	50 ± 11.79
Asia (n = 12)	100 ± 0	100 ± 0	63.9 ± 9.6	72.2 ± 23.9	75 ± 26.1	0 ± 0	63.2 ± 8.3
*P*	**–**	**–**	0.06	NS	0.057	**–**	***0.006***

* Includes partial or complete adherence; item adherence cell values represent n (%), section adherence and overall adherence cell values represent mean ± standard deviation; *P* values: italicized bold cells indicate statistical significance, values falling short of statistical significance were left in the table, non-significant values indicated by NS; – not applicable; T Treatment; O others; N North; S South; values rounded to one decimal point

Table 5 also depicts adherence by six characteristics. In terms of section adherence, collaboration with other countries or lack thereof was not associated with section adherence. Compared to abstracts by fewer (1–4) authors, authorship by more (≥ 5) authors had higher adherence for the results and discussion sections (*P* = 0.034, 0.005, respectively). Abstracts published during 2020 or after exhibited better adherence of the results section (*P* = 0.02) compared to those published ≤ 2019. Abstracts of treatment effects showed higher adherence of the results section (*P* = 0.01). As for overall adherence, abstracts with larger number of authors, published during 2020 or after, and of metanalyses were associated with higher overall adherence (*P* = 0.001, 0.04, 0.008, respectively); those from South America had lower overall adherence (*P* = 0.006); and the presence/lack of collaboration was not associated with overall adherence.

### Abstract characteristics associated with overall adherence

Table 6 shows the univariable and multivariable analyses of overall adherence. The independent variables comprised: authorship country (geographic zone), number of authors, research topic, MA or not, collaboration or not, publication period, and % of abstract word count used. In the univariate analyses, larger number of authors, MA and publication date ≥ 2020 had higher overall adherence scores (*P* = 0.001, 0.008, and 0.04, respectively). Likewise, increase in % of abstract word count used was associated with higher adherence (*P* = 0.01). No significant differences in overall adherence were observed for geographic zone, research topic, or whether the effort was a collaboration or not.

**Table 6.** Univariable and multivariable regression of overall adherence* (N = 62).

		Univariate			Multivariate			
Characteristic	Category	ß	95% CI	P	ß	95% CI	P	R^2^
Geographic zone	Asia	Referent						0.219
	Europe	3.5	−2.5; 9.5	0.25	−0.1	−6.7; 6.4	0.97	
	SA	−13.2	−26.5; 0.1	0.05	−13.2	−26.1; −0.4	***0.04***	
	NA	−3.6	−10.6; 3.4	0.31	−5.6	−12.8; 1.6	0.13	
	Africa	−7.2	−15.5; 1.0	0.09	−6.6	−15.4; 2.2	0.14	
Author group	≥ 5	Referent						
	1–4	−8.1	−12.5; −3.7	***0.001***	−4.2	−9.3; 0.9	0.11	0.184
Research Topic	Others	Referent						0.013
	Treatment	2.1	−2.71;7.0	0.38	1.3	−3.7; 6.3	0.59	
Meta-analysis	Yes	Referent						0.113
	No	−8.6	−14.9; −2.4	***0.008***	−4.8	−10.7; 1.1	0.11	
Collaboration	No	Referent						
	Yes	0.2	−4.7; 5.1	0.93	−5.0	−9.9; −0.1	***0.046***	0.001
Publication period	≥ 2020	Referent						
	≤ 2019	−4.9	−9.7; −0.2	***0.04***	−3.3	−7.7; 1.1	0.14	0.068
% of abstract word count used		0.20	0.1; 0.4	***0.01***	0.1	−0.1; 0.2	0.26	

*Includes partial/complete adherence SA, South America; NA North America, CI confidence interval; italicized bold cells indicate statistical significance; values rounded to one decimal point

For the multivariable analyses, controlling for all other characteristics, abstracts of authors from a single country (i.e. no collaboration) were associated with higher overall adherence (*P* = 0.046), while abstracts from South America were associated with lower adherence (*P* = 0.04). Hence, these were the only two independent predictors of overall adherence. Whilst an increase of 10% abstract word count used was associated with 0.9% higher overall adherence, the relationship was not significant. R^2^ of the various characteristics ranged from 0.001 to 0.219; and R^2^ of whole model was 0.411, indicating that about 41% of the variance in overall adherence was explained by the seven characteristics included in the model.

## Discussion

Findings of SR/MAs guide patient care and future research [27,28]. Urologists need to sieve out relevant research from a pool of published literature to remain updated about practice. Whilst SRs may generate estimates that are less biased [29], findings should be reported clearly and completely in the abstracts [23]. Appraisals of the reporting quality of abstracts of SR/MAs in urology are non-existent. Hence, we were unable to directly compare our findings with similar appraisals in urology.

Our main findings show that adherence to the 12 PRISMA-A items was: 100% adherence, 2 items (title, objectives); 80% to <100%, 5 items (interpretation, included studies, synthesis of results, eligibility criteria, information sources); <80–40% 2 items (description of effect, strengths/ limitations of evidence); 1.6–0% adherence, 3 items (risk of bias, funding/ conflict of interest, registration). For overall adherence, the multivariable analyses showed that, controlling for all characteristics, the only two independent predictors of overall adherence were that single-country authorship (i.e. no collaboration) was associated with higher overall adherence (*P* = 0.046), while abstracts from South America were associated with lower adherence (*P* = 0.04).

In connection with overall adherence to PRISMA-A, our mean overall adherence was 62.8%, agreeing with an assessment of SR abstracts of medical interventions where on average, abstracts reported 60% of the PRISMA-A items [18]. More than two-thirds of abstracts we appraised (66%) demonstrated moderate-to-good overall adherence (>50% to < 75%), while 18% had high overall adherence (70%), supporting that the majority of abstracts of SR/MAs in e.g. dentistry journals were of moderate quality [30]. Others observed that although the introduction of the PRISMA-A checklist enhanced the reporting of abstracts in orthodontic SRs (from 48% to 59%), compliance could still be bettered [3]. Below, we compare our findings by item with the published literature.

As for title and objectives, we observed 100% adherence to both, in agreement with others where 100% and 87.5% of abstracts adequately reported title and objectives, respectively [30]. Others noted that study objectives were well reported in SR abstracts [18]. We found that the eligibility criteria also displayed high adherence (87.1%), supporting that 85.42% of abstracts adhered to PRISMA-A when reporting eligibility criteria [30]. Whilst other research did not provide the reasons for <100% adherence, the reasons we found behind partial reporting of objectives were omissions in either the comparator, outcome/s, population, or a combination of population and comparator, rather than omission of the intervention; and for eligibility criteria, it was invariably because of omission of type of studies. Such omissions are feasible to rectify and would raise the abstracts’ quality.

In terms of risk of bias, only 1.6% of abstracts we inspected mentioned any type of risk of bias assessed by the authors. A major inadequacy when appraising the reporting of PRISMA-A was the reporting of risk of bias (41.67% of studies), as the least frequently reported items included risk of bias assessment (12%) [18,30]. Due to the large numbers of SRs published, bias appraisal is important in urology as in other specializations [29]. Bias can be related to the actual studies included in a SR; or to reporting bias (outcome reporting bias) [31]. Notwithstanding, our observed lack of bias assessment reporting does not necessarily translate to that bias assessment was not actually undertaken by the authors, as it could have been reported in the manuscript rather than the abstract. Even so, such omissions might deter busy clinicians from retrieving full texts, thus missing important developments. Reporting quality in abstracts should be similar to the manuscript [23], particularly that checklists are available (e.g. PRISMA-A).

The current study found that adherence of the strengths/limitations of the evidence item was 43.5%, supporting that such reporting needs attention [18]. In agreement, < 30% of orthodontics SR/MAs’ abstracts were compliant with the strengths/limitations of the evidence item [3]. For some abstracts that we appraised, we could implicitly conclude some limitations, rather than those being overtly declared by the authors. A SR’s value is in what was done, what was found, and the reporting clarity. Explicit mention of strengths/limitations helps readers to assess constraints and their influence on practice.

In terms of registration, adherence was 0% in the current study, congruent with others where protocol registration was among the least frequently reported items (2%) [18]; and that the lack of reporting of registration (4.17%) was a major inadequacy in SR/MAs abstract appraisals [30]. Others observed the low adherence of SR/MAs to the PRISMA-A checklist of the registration item [3]. Registered reviews offer better quality and conduct and reporting completeness compared to non-registered ones, and *a priori* registration of SR protocols could boost transparency, minimize outcome reporting bias, and reduce improper spending of research funds due overlaps between SRs [32–34].

As regards to funding, adherence was 0% in the present study, exactly similar to other evaluations [30]. Among 200 SR abstracts, the least frequently reported items included funding source (1%) [18]. Declaring sources of funding or lack thereof are critical, and a funding agency with no financial interest in the study assures reliable findings [30].

As for the item of the description (direction, size) of the effect in terms meaningful to clinicians/patients (PRISMA-A results subsection), we observed that 61.3% were adherent, supporting that 45.9% of dentistry abstracts did not provide effect size [30]. Others noted similar findings, where ‘fewer than half of abstracts described results in terms meaningful to patients and clinicians’ [18]. SR abstracts seldom report absolute measures [35], and relative measures could overestimate efficacy [36]. Abstracts also need to note the differences between numerical statistical significance and worthy clinical importance, and advise readers if observed effects translate into meaningful change for patients [18].

A related issue is that the effect description could entail ‘negative’ findings. Non-statistically significant findings might point to a lack of precision, or equivalence/non-inferiority between interventions, and terms like ‘no evidence of effect’ do not differentiate between these two reasons [18]. There is hesitation to publish negative results [37], despite philosophical and practical support [38]. Although researchers value publication of ‘negative’ results, they frequently do not publish their own, probably due to lack of time and that findings might not be highly cited [39].

Regarding the associations between abstract characteristics and completeness of reporting, our multivariable analyses showed that, after controlling, the two independent predictors of overall adherence were that single-country authorship (i.e. no collaboration) was associated with higher adherence, while abstracts from South America had lower adherence. Others observed no association between abstracts’ quality and number of authors, country, journals, publication year, word count, or study focus [30]. We found no significant relationship between % of word count used in the abstract and overall adherence, although a 10% increase in word count used increased overall adherence by 0.9% (P = 0.26). Elsewhere, the abstract’s word counts explained 13% of the PRISMA-A score variance, and for every 50 additional words in the abstract, the score was forecasted to rise 0.6 points, concluding that inadequate reporting cannot be attributed to word count restrictions alone [18].

The present study excluded three unstructured abstracts. Applying PRISMA-A is best with structured abstracts, as the checklist is categorized into six sections. Our exclusions agree with a study where the review team decided to include/exclude given articles based on whether the abstract satisfied the prespecified criteria of the tool employed [40]. Some journals detail the abstract’s structure, others offer a word limit; *AJU* specifies and requires both. Structured abstracts provide higher quality and more information, could be easier to read, search, and facilitate peer review, and are welcomed by readers/authors [41–43]. Structured abstracts had significantly higher quality compared to unstructured ones; and change in abstract format from unstructured to structured affected quality positively [41,44].

## Implications

Published SRs are about > 8000 per year, with financial implications related to unclear abstracts [40,45]. Health-care providers needing to sustain up-to-date practice have to rapidly recognize the most relevant literature. The suggestions below might contribute to well-designed, well-reported abstracts of SR/MAs in urology. This would assist clinicians to interpret the findings and appraise the strengths/limitations of such evidence [23,46].

For Journals, editorial teams could: 1) Endorse structured abstract format and PRISMA recommendations if it is not already the case; 2) Ensure SR/MAs manuscripts are processed only after structured abstracts with specified word count following the PRISMA-A are submitted; and, 3) Provide direction to reviewers and authors about adherence to PRISMA-A checklist. Regarding health-care providers, appropriate conduct of SRs demands application of a series of steps and specific skills, sometimes deficient among practitioners [47,48]. Hence, adequate training to undertake SR/MAs is required. For authors, they need to: 1) Follow the journal instructions in relation to abstract structure and word count; 2) Report findings in accordance with the PRISMA-A; and 3) Include limitations e.g. risk of bias and heterogeneity estimates of included primary studies; or for MAs, present numerical data (e.g. CI, p values, etc.). Pertaining to funding agencies, a requisite for funding SR/MAs applications could be endorsement of PRISMA/ PRISMA-A checklists.

Inspection of the items with partial reporting (Table 3) suggested that feasible efforts could considerably raise the reporting completeness of SR/Ms abstracts. These include: for objectives, reporting the comparator, outcome/s, population; for eligibility criteria, reporting the type of studies; for included studies, reporting type of studies and participants; for information sources, reporting search dates; for synthesis of results, reporting number of participants for each outcome and benefits/harms for the outcome/s; and for strengths/ limitations of evidence, reporting the strengths.

This study has limitations. We assessed one journal and generalizations to urology journals need to be cautious. In addition, evaluating some PRISMA-A items can be more subjective than others [18], e.g. appraisal of the title, names and database search dates is straightforward; appraisal of other PRISMA-A checklist items could be more challenging.

Nevertheless, the strengths of the study are that it assessed the abstracts of all SR/MAs published in one journal. Data extraction and scoring was intentionally performed by two research team members working simultaneously together on each abstract and reaching consensus for each PRISMA-A checklist item. Whilst this process is more labor-intensive/time-consuming than the conventional extraction and scoring by a single researcher, it ensured better consensual calibration of each abstract and not only a sample of abstracts as traditionally undertaken in such appraisals [3,18,23,30]. Unlike others who examined only one potential characteristic (abstract word count) to explain PRISMA-A scores variation, we appraised seven potential characteristics (number of authors, country, publication period, topic of study, collaboration, whether abstract was for SR or for SR+MA, and abstract word count). The current study could be the first to undertake such in-depth appraisal of the quality of abstracts of SR/MAs in urology whilst also exploring any associations between abstract characteristics and overall adherence. Abstracts play a key role in disseminating the findings of SR/MAs, and the current appraisal emphasizes areas for enhancements in order to stimulate interpretation and utility.

## Conclusions

Completeness in the reporting of abstracts of SR/MAs renders it easier for readers to reliably appraise the findings. This study appraised the adherence levels by item and section of the PRISMA-A checklist, as well as overall adherence. Some items exhibited 100% adherence (title, objectives), while others displayed very low adherence (risk of bias, funding/ conflict of interest, registration). Only two independent predictors of overall adherence were observed: single-country authorship (i.e. no collaboration) was associated with higher overall adherence, while abstracts from South America were associated with lower adherence. Some feasible efforts would enhance the completeness of the SR/MAs abstracts considerably. These include raising authors’ awareness about reporting comparator/s, outcome/s, population, search dates, type of studies, results for the outcome/s (benefits and harms), number of participants, strengths of the evidence, as well as risk of bias, funding, and registration. There is room for improving the quality of reporting SR/MAs in urology. Journals should consider having specific criteria-based checklists before review and publication of manuscripts to ensure the high reporting quality.

## Appendix 1

01- Radiation therapy compared to radical prostatectomy as first-line definitive therapy for patients with high-risk localised prostate cancer: An updated systematic review and meta-analysis. Aydh A, Motlagh RS, Abufaraj M, et al. Arab J Urol. 30 March 2022;20(2):71–80.

02- The health-related quality of life in patients with prostate cancer managed with active surveillance using the Expanded Prostate Cancer Index Composite survey: Systematic review and meta-analysis. Abdelhafez A, Hosny K, El-Nahas AR, Liew M. Arab J Urol. 27 February 2022;20(2):61–70.

03- The effect of neoadjuvant chemotherapy among patients undergoing radical cystectomy for variant histology bladder cancer: A systematic review. Alvarez-Maestro M, Chierigo F, Mantica G, et al. Arab J Urol. 7 November 2021;20(1):1–13.

04- Fresh vs frozen testicular sperm for assisted reproductive technology in patients with non-obstructive azoospermia: A systematic review. Amer M, Fakhry E. Arab J Urol. 6 July 2021;19(3):247–254.

05- Long-term consequences of sexually transmitted infections on men’s sexual function: A systematic review.Henkel R. Arab J Urol. 7 July 2021;19(3):411–418.

06- An update on the treatment of premature ejaculation: A systematic review. Saleh R, Majzoub A, Abu El-Hamd M. Arab J Urol. 4 August 2021;19(3):281–302. doi: 10.1080/2090598X.2021.1943273. PMID: 34,552,780; PMCID: PMC8451625.

07- Current updates and future perspectives in the evaluation of azoospermia: A systematic review. Punjani N, Kang C, Lamb DJ, Schlegel PN. Arab J Urol. 22 July 2021;19(3):206–214.

08- Tissue and sperm handling before assisted reproductive technology (ART): A systematic review. Ambar RF, Gava MM, Ghirelli-Filho M, et al. Arab J Urol. 22 July 2021;19(3):238–246.

09- The impact of COVID-19 on the male reproductive tract and fertility: A systematic review. Sengupta P, Leisegang K, Agarwal A. Arab J Urol. 9 August 2021;19(3):423–436.

10-Medical management of non-obstructive azoospermia: A systematic review. Alkandari MH, Zini A. Arab J Urol. 24 July 2021;19(3):215–220.

11-Varicocele treatment in non-obstructive azoospermia: a systematic review. Jensen S, Ko EY. Arab J Urol. 26 July 2021;19(3):221–226.

12-Peyronie’s disease – outcomes of collagenase clostridium histolyticum injection: A systematic review. Mefford AT, Raheem O, Yafi FA, Alzweri LM. Arab J Urol. 16 August 2021;19(3):363–369.

13-A systematic review on the latest developments in testosterone therapy: Innovations, advances, and paradigm shifts. Al-Zoubi RM, Yassin AA, Alwani M, et al. Arab J Urol. 8 August 2021;19(3):370–375.

14- The usefulness of elastography in the evaluation and management of adult men with varicocele: A systematic review. Bello JO, Bhatti KH, Gherabi N, et al. Arab J Urol. 16 September 2021;19(3):255–263.

15- Alternative medicine and herbal remedies in the treatment of erectile dysfunction: A systematic review. Leisegang K, Finelli R. Arab J Urol. 11 June 2021;19(3):323–339.

16- Vitamins as primary or adjunctive treatment in infertile men with varicocele: A systematic review. Tsampoukas G, Gkeka K, Dellis A, et al. Arab J Urol. 29 May 2021;19(3):264–273.

17- The effectiveness of psychological interventions alone, or in combination with phosphodiesterase-5 inhibitors, for the treatment of erectile dysfunction: A systematic review. Atallah S, Haydar A, Jabbour T, et al. Arab J Urol. 27 May 2021;19(3):310–322.

18- Novel methods to enhance surgical sperm retrieval: a systematic review. Kresch E, Efimenko I, Gonzalez D, et al. Arab J Urol. 18 May 2021;19(3):227–237.

19- Drug-delivering devices in the urinary tract: A systematic review. Kallidonis P, Adamou C, Castillo SV, et al. Arab J Urol. 3 March 2021;19(2):191–204.

20- Music reduces patient-reported pain and anxiety and should be routinely offered during flexible cystoscopy: Outcomes of a systematic review. Gauba A, Ramachandra MN, Saraogi M, Geraghty R, et al. Arab J Urol. 3 March 2021;19(4):480–487.

21- The role of radiological surveillance in the conservative management of incidental small testicular masses: A systematic review. Brown D, Tsampoukas G, Popov EP, Aldin Z, et al. Arab J Urol. 11 February 2021;19(2):179–185.

22- The association between sarcopenia and bladder cancer-specific mortality and all-cause mortality after radical cystectomy: A systematic review and meta-analysis. Ibilibor C, Psutka SP, Herrera J, et al. Arab J Urol. 16 January 2021;19(1):98–103.

23-Performance of fluoro-2-deoxy-D-glucose positron emission tomography-computed tomography imaging for lymph node staging in bladder and upper tract urothelial carcinoma: a systematic review.Aydh A, Abufaraj M, Mori K, et al. Arab J Urol. 10 December 2020;19(1):59–66.

24- Prognostic impact of perioperative blood transfusions on oncological outcomes of patients with bladder cancer undergoing radical cystectomy: A systematic review. Volz Y, Eismann L, Pfitzinger PL, et al. Arab J Urol. 10 December 2020;19(1):24–30.

25- Frailty impact on postoperative complications and early mortality rates in patients undergoing radical cystectomy for bladder cancer: a systematic review. Ornaghi PI, Afferi L, Antonelli A, et al. Arab J Urol. 2 November 2020;19(1):9–23.

26- Abi Chebel J, Sarkis J, El Helou E, et al. Minimally invasive simple prostatectomy in the era of laser enucleation for high-volume prostates: A systematic review and meta-analysis. Arab J Urol. 11 August 2020;19(2):123–129.

27- Lymph node dissection for upper tract urothelial carcinoma: A systematic review. Chan VW, Wong CHM, Yuan Y, Teoh JY. Arab J Urol. 27 July 2020;19(1):37–45.

28-Inflammatory bowel disease and prostate cancer risk: A systematic review. Haddad A, Al-Sabbagh MQ, Al-Ani H, et al. Arab J Urol. 19 May 2020;18(4):207–212.

29-The effectiveness of multiparametric magnetic resonance imaging in bladder cancer (Vesical Imaging-Reporting and Data System): A systematic review. Carando R, Afferi L, Marra G, et al. Arab J Urol. 1 March 2020;18(2):67–71.

30- A systematic review and meta-analysis of clinical and functional outcomes of artificial urinary sphincter implantation in women with stress urinary incontinence. Barakat B, Franke K, Hijazi S, et al. Arab J Urol. 4 February 2020;18(2):78–87.

31-Antibiotic therapy in patients with high prostate-specific antigen: Is it worth considering? A systematic review. Taha DE, Aboumarzouk OM, Koraiem IO, Shokeir AA. Arab J Urol. 25 October 2019;18(1):1–8.

32-Trends in quality of life reporting for radical cystectomy and urinary diversion over the last four decades: A systematic review of the literature. Rangarajan K, Somani BK. Arab J Urol. 14 April 2019;17(3):181–194.

33- Oxidative stress and sperm function: A systematic review on evaluation and management. Dutta S, Majzoub A, Agarwal A. Arab J Urol. 24 April 2019;17(2):87–97

34-Urethral diverticulum: A systematic review. Greiman AK, Rolef J, Rovner ES. Arab J Urol. 8 April 2019;17(1):49–57.

35- Mechanical inserts for the treatment of faecal incontinence: A systematic review. Buono K, Davé-Heliker B. Arab J Urol. 8 April 2019;17(1):69–76.

36- Management of vaginal mesh exposure: A systematic review. Bergersen A, Hinkel C, Funk J, Twiss CO. Arab J Urol. 4 April 2019;17(1):40–48.

37- Systematic review of lower urinary tract symptoms occurring with pelvic organ prolapse. Cameron AP. Arab J Urol. 3 April 2019;17(1):23–29.

38-Update on vesicovaginal fistula: A systematic review. El-Azab AS, Abolella HA, Farouk M. Arab J Urol. 4 April 2019;17(1):61–68.

39- Oral desmopressin in nocturia with benign prostatic hyperplasia: A systematic review of the literature. Taha DE, Aboumarzouk OM, Shokeir AA. Arab J Urol. 25 July 2018;16(4):404–410.

40- Robotic stone surgery – Current state and future prospects: A systematic review. Müller PF, Schlager D, Hein S, et al. Arab J Urol. 2 November 2017;16(3):357–364.

41- Complications in robotic urological surgeries and how to avoid them: A systematic review. Tourinho-Barbosa RR, Tobias-Machado M, Castro-Alfaro A, et al. Arab J Urol. 14 December 2017;16(3):285–292.

42-Robot-assisted radical cystectomy with intracorporeal urinary diversion – The new ‘gold standard’? Evidence from a systematic review. Lobo N, Thurairaja R, Nair R, et al. Arab J Urol. 11 April 2018;16(3):307–313.

43-Laparoscopic and hand-assisted laparoscopic donor nephrectomy: A systematic review and meta-analysis. Broe MP, Galvin R, Keenan LG, Power RE. Arab J Urol. 7 July 2018;16(3):322–334.

44- Laparoscopic and hand-assisted laparoscopic donor nephrectomy: A systematic review and meta-analysis. Broe MP, Galvin R, Keenan LG, Power RE. Arab J Urol. 7 July 2018;16(3):322–334.

45- Selective embolisation for intractable bladder haemorrhages: A systematic review of the literature. Taha DE, Shokeir AA, Aboumarzouk OA. Arab J Urol. 2 March 2018;16(2):197–205.

46- Prediction of male infertility by the World Health Organization laboratory manual for assessment of semen analysis: A systematic review. Patel AS, Leong JY, Ramasamy R. Arab J Urol. 2 November 20,170;16(1):96–102.

47-Role of sperm DNA fragmentation in male factor infertility: A systematic review. Cho CL, Agarwal A. Arab J Urol. 6 December 2017;16(1):21–34.

48-Varicocele management for infertility and pain: A systematic review. Lundy SD, Sabanegh ES Jr. Arab J Urol. 14 December 2017;16(1):157–170.

49- Advances in sperm retrieval techniques in azoospermic men: A systematic review. Shah R, Gupta C. Arab J Urol. 26 December 2017;16(1):125–131.

50- Systematic review of hormone replacement therapy in the infertile man. El Meliegy A, Motawi A, El Salam MAA. Arab J Urol. 30 December 2017;16(1):140–147.

51- Systematic review of hormone replacement therapy in the infertile man. El Meliegy A, Motawi A, El Salam MAA. Arab J Urol. 30 December 2017;16(1):140–147.

52- Update on the proteomics of male infertility: A systematic review. Panner Selvam MK, Agarwal A. Arab J Urol. 29 December 2017;16(1):103–112.

53- A systematic review on sperm DNA fragmentation in male factor infertility: Laboratory assessment.

Panner Selvam MK, Agarwal A. Arab J Urol. 17 January 2018;16(1):65–76.

54-A systematic review on the genetics of male infertility in the era of next-generation sequencing. Robay A, Abbasi S, Akil A, El-Bardisi H, Arafa M, Crystal RG, Fakhro KA. Arab J Urol. 14 February 2018;16(1):53–64.

55-Critical appraisal of literature comparing minimally invasive extraperitoneal and transperitoneal radical prostatectomy: A systematic review and meta-analysis. Kallidonis P, Rai BP, Qazi H, et al. Arab J Urol. 31 August 2017;15(4):267–279.

56- The effectiveness of ureteric metal stents in malignant ureteric obstructions: A systematic review. Kallidonis P, Kotsiris D, Sanguedolce F, et al. Arab J Urol. 16 October 2017;15(4):280–288.

57- Trends of intervention for paediatric stone disease over the last two decades (2000–2015): A systematic review of literature. Pietropaolo A, Proietti S, Jones P, et al. Arab J Urol. 2 November 20,170;15(4):306–311.

58- Laparoscopic vs robotic nephroureterectomy: Is it time to re-establish the standard? Evidence from a systematic review. Stonier T, Simson N, Lee SM, et al. Arab J Urol. 16 June 2017;15(3):177–186.

59- Photodynamic diagnosis in upper urinary tract urothelial carcinoma: A systematic review. Osman E, Alnaib Z, Kumar N. Arab J Urol. 6 March 2017;15(2):100–109.

60- Medical expulsive therapy for ureteric stones: Analysing the evidence from systematic reviews and meta-analysis of powered double-blinded randomised controlled trials. Amer T, Osman B, Johnstone A, et al. Arab J Urol. 18 April 2017;15(2):83–93.

61- Antibiotic prophylaxis and its appropriate timing for urological surgical procedures in patients with asymptomatic bacteriuria: A systematic review. Ramos JA, Salinas DF, Osorio J, Ruano-Ravina A. Arab J Urol. 10 June 2016;14(3):234–9.

62- Squamous cell carcinoma of the urinary bladder: Systematic review of clinical characteristics and therapeutic approaches. Martin JW, Carballido EM, Ahmed A, et al. Arab J Urol. 1 August 2016;14(3):183–91.

63- What is better in percutaneous nephrolithotomy – Prone or supine? A systematic review. Mak DK, Smith Y, Buchholz N, El-Husseiny T. Arab J Urol. 4 March 2016;14(2):101–7.

64- Innovative trends and perspectives for erectile dysfunction treatment: A systematic review. Ismail EA, El-Sakka AI. Arab J Urol. 18 May 2016;14(2):84–93.

65- Holmium laser enucleation versus simple prostatectomy for treating large prostates: Results of a systematic review and meta-analysis. Jones P, Alzweri L, Rai BP, et al. Arab J Urol. 2016 Mar;14(1):50–8.

66- The incidence of erectile dysfunction after pelvic fracture urethral injury: A systematic review and meta-analysis. Blaschko SD, Sanford MT, Schlomer BJ, et al. Arab J Urol. 2015 Mar;13(1):68–74.
